# A training program for researchers in population neuroimaging: Early experiences

**DOI:** 10.3389/fnimg.2022.896350

**Published:** 2022-08-25

**Authors:** Caterina Rosano

**Affiliations:** Department of Epidemiology, Graduate School of Public Health, University of Pittsburgh, Pittsburgh, PA, United States

**Keywords:** population neuroscience, Alzheimer's Disease, geroscience, epidemiology, neuroimaging

## Abstract

Recent advances in neuroimaging create groundbreaking opportunities to better understand human neurological and psychiatric diseases, but also bring new challenges. With the advent of more and more sophisticated and efficient multimodal image processing software, we can now study much larger populations and integrate information from multiple modalities. In consequence, investigators that use neuroimaging techniques must also understand and apply principles of population sampling and contemporary data analytic techniques. The next generation of neuroimaging researchers must be skilled in numerous previously distinct disciplines and so a new integrated model of training is needed. This tutorial presents the rationale for such a new training model and presents the results from the first years of the training program focused on population neuroimaging of Alzheimer's Disease. This approach is applicable to other areas of population neuroimaging.

## Introduction

Neuroimaging is a powerful tool to study neurological and psychiatric conditions; it can help discover novel targets for prevention and treatment, and can provide biomarkers to monitor a disease over time and in response to treatment (Young et al., 2020). The rapid growth of neuroimaging technologies and other methods of human health assessment present innumerable opportunities for discovery (Calhoun et al., 2021; Singh et al., 2022), but also precipitate the need to change training models for the next generation of scientists. Ever-expanding multimodal neuroimaging techniques, combined with the rapid growth of supporting basic neurosciences, and increased feasibility of multi-systemic data acquisition in larger sample sizes, require that the next generation of neuroimaging researchers have knowledge and skills from multiple disciplines.

Traditionally, neuroimaging researchers focus on characterizing the structure and function of the brain in highly selected, homogenous clinical or volunteer populations. This otherwise excellent training risks overlooking the complexity of the aging process, and the epidemiological perspectives on the influence that sampling procedures exerts on their findings and reproducibility. For example, early life and development, behavior, culture, neighborhood, and environment, alone and together with demographics, can influence brain function and structure. An unintended consequence of such limitations is that most neuroimaging studies examine small and potentially unrepresentative samples, sometimes called WEIRD -Western Educated Industrialized Rich Democracies (Henrich et al., 2010; Hwang, 2022), with findings that do not always generalize to the rest of the world (Marek et al., 2022).

This tutorial provides evidence that the integration of neuroimaging with population science, data analytical methods, and basic and clinical sciences, has a strong potential to address the limitations of current training models. In fact, such high-level integration across fields can advance our understanding of the etiology and pathophysiology of brain diseases. This notion is based on the emerging field of population neuroscience, which can be conceptualized as a “marriage” between the disciplines of populations science and neuroscience (Paus, 2010; Falk et al., 2013; Ganguli, 2022).

This tutorial is intended to provide the rationale, framework, processes, and preliminary outcomes of a novel approach to multidisciplinary training in population neuroimaging, based on the first few years of the T32 training program in Population Neuroscience of Alzheimer's Disease (PNA). The PNA promotes a new model of training and research in brain aging that moves beyond a focus on an individual brain disease alone, toward a multidisciplinary approach built on knowledge and skills in neuroimaging, epidemiology, data sciences and gerontology. The PNA training program is open to a broad group of trainees who might enter the program with expertise in any one (or more) of the fields of neuroscience (includes neuroimaging), population science, data science, and gerontology (Paus, 2010; Falk et al., 2013; Ganguli et al., 2018). Since this special issue is dedicated to population neuroimaging, the focus is on how training programs can prepare the next generation of neuroimaging researchers in population and data sciences. A multidisciplinary program to train population neuroimaging researchers requires collaborations across academic departments and active multidisciplinary mentoring teams. Trainees who enter such multidisciplinary training programs may come from a wide variety of prior disciplines, so the training plan for each must be individualized based on competencies already achieved and those that need to be acquired. Advancement in research increasingly require a team science approach; accordingly, a training program dedicated to population neuroimaging will provide unique opportunities to collaborate with top scientists in their fields of expertise (i.e., epidemiology, neuroscience, data science, gerontology). By leveraging the specific strengths of these researchers and by having them collaborate with trainees, the quality of the work will further improve. Hence, skills in collaboration should also actively be trained.

We recognize that terminology from one discipline may be unfamiliar or ambiguous to scholars from other disciplines, and multidisciplinary programs must explicitly work to develop a shared knowledge base of terms and concepts. For this reason, Table 1 has a glossary of terms; it would be appropriate for multidisciplinary programs focusing on other topics to develop their own glossary.

**Table 1:** Glossary.

**Term**	**Definition**
Incidence	The rate at which new cases of a disease develop in the population at risk over a defined period of time.
Prevalence	Proportion of cases in the population at a given time. It is a function both of incidence and of duration, that is how long people with the disease remain in the population.
ADRD	Alzheimer's Disease and Related Dementias.
Neuroscience	Disciplines measuring the health of the central nervous system. It includes neuroimaging, psychology, neurobiology among others.
Population sciences	Disciplines studying the causes, manifestations and distribution of a disease in populations to uncover patterns, trends, and outcomes that may be applicable to the general population. Knowledge of population sciences is essential to design studies to identify groups of people who are at high risk for developing a certain condition and to implement, and assess approaches to effectively prevent disease and improve quality of life in the population.
Gerontology	Includes clinical and physiological processes of aging, such as diabetes, hypertension etc, as well as basic biological mechanisms, for example genomics, proteomics, metabolomics. Gerontologists can be researchers with clinical (medical, nursing, physical therapy degree) and/or research degrees (PhD, DrPh), or clinicians with expertise in ADRD (neurologists, psychiatrists).
Data science	A host of quantitative approaches, computational science, machine learning, regression tree algorithms, that produce risk models applied to biomedical problems. It includes computational biology, bioinformatics, mathematics, engineering, and biostatistics, among others.
Multi-morbidities	Coexistence of 1 or more chronic diseases (diabetes mellitus, stroke, hypertension, cancer, chronic obstructive pulmonary diseases, heart disease, osteoarthritis/ chronic pain) and/or risk factors (higher blood pressure/glucose, dyslipidemia, etc.).

## The story of Alzheimer's Disease through the lens of population science

**Figure 1:**
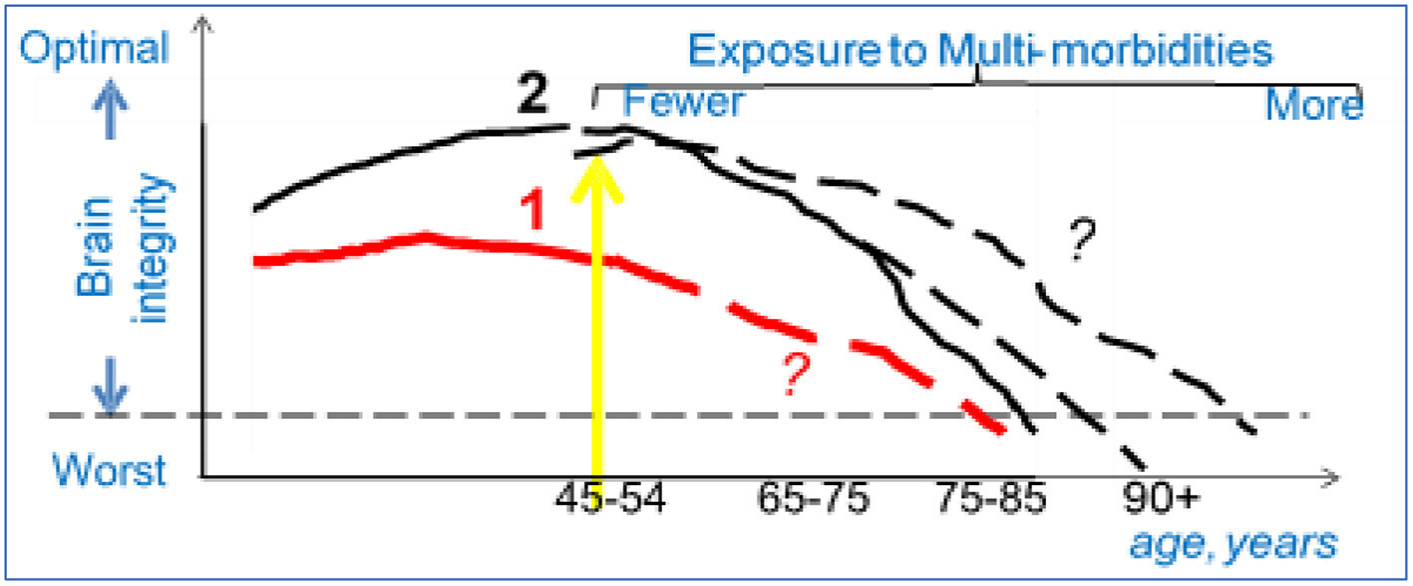
Population science to study AD: an example. Group 1 (red line): Patients exposed to chronic diseases in childhood (T1D) or to trauma/vascular accidents in adulthood (TBI, heart attack) develop lower brain integrity earlier in life (worse brain structure and/or function). With newer therapies the life span of these patients is extending (yellow arrow), but the trajectories of decline in brain integrity for these new age group are not clear (dashed line, question marks). Group 2 (black line): Adults who enter early old age (>55), are exposed to conditions that can reduce brain integrity either directly (e.g., stroke) or indirectly as the result of co-morbidities (e.g., diabetes). Better therapies for these conditions are expanding the lifespan, but the trajectories of decline in brain integrity for this new age group is not clear (dashed lines, question marks).

It is well-known that chronological age and age-related factors affect dementia risk. However, two emerging phenomena are changing the apparent phenotype of brain aging and that will influence how we identify the population at risk for AD in the future (Figure 1). The first phenomenon relates to longer life span. People with childhood or midlife chronic disease, such as stroke or type 1 diabetes (group 1) and older adults in general (group 2), live longer now than ever before. Extended lifespan contributes to greater risk of AD, because older chronological age is a risk factor for dementia; in other words, the size of the at-risk population is becoming larger. Extended lifespans would mean that there is in increase in conditions that could damage the brain either directly (cerebrovascular and neurodegenerative pathology) or indirectly, *via* dysregulation of biological factors, including epigenetic factors, and chronic multi-morbidities (Kennedy et al., 2014; Austad, 2016). Multi-morbidities can compromise brain integrity through complex pathways, including diabetes, hypertension, chronic obstructive pulmonary disease, heart disease, depression, as well as higher levels of lipids, blood pressure, glucose, oxidative stress, and inflammatory factors. In other words, this remarkable increase in lifespan is accompanied by only modest decreases in multi-morbidities and modest increases in healthspan (the number of years one is expected to remain functionally independent).

Due to the discrepancy between healthspan and lifespan, the prevalence of ADRD will remain constant or possibly even increase over time. Even with lower incidence in many disease (e.g., stroke), there will be an increase in the number of individuals being exposed to morbidities typical of older chronological age; that is because prevalence of a condition is related to both duration and incidence (Prevalence = duration X incidence). Please refer to Table 1 and to the excellent tutorial in epidemiology (Jacob and Ganguli, 2016) for the epidemiologic concepts of incidence and prevalence. In addition to leading to growing numbers of adults at risk for ADRD, these phenomena will lead to a highly heterogeneous population of older adults, who no longer fit into a “one size fit all” category, and will require dedicated recruitment and assessment techniques. Recent demographic transitions further contribute to increase the heterogeneity of the aging population. First, there are greater numbers of racial/ethnic minorities with diverse exposure histories who are aging. Secondly, changes in lifespan and health-span affect men and women differently, with consequences for the distribution of disease burden by sex.

Why should neuroimaging researchers care for these epidemiological phenomena? We will need novel strategies to assess CNS integrity, to create accurate prediction models about brain changes over time (dashed lines, Figure 1). For example, neuroimaging methods to quantify brain integrity in adults aged 90 years old are likely to be different from those in Type 1 diabetics in their 50's.

Secondly, while each factor alone (e.g., chronological age, multi-morbidities, history of chronic diseases, demographic transitions in ethnicity) contributes to ADRD, the interaction among these factors likely influence ADRD in complex ways. For example, while it is reasonable that brain integrity would decline and dementia prevalence increase over time, it cannot be assumed that chronological years and years spent with a chronic condition (e.g., Type 1 Diabetes or Traumatic Brain Injury in childhood/young adulthood) would have linearly additive effects on brain integrity. Neither can we expect that effects would be similar for men and women, different ethnicities, or for different patterns of multi-morbidities. Another challenge that awaits neuroimaging researchers is the need to integrate neuroimaging data with other physiological and health-related data. With greater recent technological advancements, it is easier to collect more data in more people and to examine a wide variety of data at low cost (e.g., saliva swab). These studies generate massive volumes of data (“Big Data”) and require investigators to apply novel and sophisticated data processing and analytic techniques.

There are likely to be dementia mechanisms waiting to be discovered if the relationship between chronic diseases and neurodegeneration can be explored in a systematic and multidisciplinary manner. Accordingly, there are many novel questions in ADRD that modern population neuroimaging researchers can now address: how rapidly will brain integrity decline for these “new” older adults; will rapidity of decline vary for different age, sex, ethnic subgroups, or patterns of comorbid conditions? Will these factors affect decline in different aspects of brain integrity (e.g., blood flow, atrophy) or behavior (e.g., attention, memory)? What factors could delay or accelerate the progression of brain abnormalities and will they differ across age groups, racial, or patients' subgroups? For example, an editorial entitled “Age, Alzheimer's Disease, and the Big Picture” (Ganguli and Rodriguez, 2011) noted the epidemiologic evidence that most known risk factors for ADRD exert their effects before age 85–90, although incidence continues to increase after that. Thus, if different underlying mechanisms are at play for different age groups, different prevention/intervention strategies may be needed in different age groups.

To address these challenges, neuroimaging researchers intending to conduct etiological research in ADRD, must have a clear grasp of heterogeneity within the aging population as a whole and also within the samples they choose to study. They must then be able to design and interpret their studies accordingly. Understanding these principles is not only critical to eventually producing personalized interventions, it is also the foundation of research that is reproducible and rigorous. In this regard, there is a paramount need to train scientists on epidemiological approaches to maximize rigor and reproducibility (Ioannidis, 2017; Munafò et al., 2017).

## Why train neuroimaging researchers in population and data sciences?

Novel training programs in population neuroimaging must develop a compelling rationale for why scholars need unique knowledge and skills in its topic area, and ensure that standard training in neuroimaging from a departmental program would not already meet these needs. The PNA training program is specifically focused on dementia, but similar issues exist for multidisciplinary programs that focus on other topics. In general, a multidisciplinary program to train neuroimaging researchers studying the cause, presentation, and course of a brain disease should address: (1) the effects of demographic (age, sex, race, but also ethnicity, culture, education, development, environment), and biological factors (genetics, epigenetics, proteomics, or other biological/physiological processes); (2) strategies to identify the population at risk for the condition of interest; and (3) adequate analytical tools to integrate these data, account for complex interactions, draw interpretations that are biologically plausible, and generate novel hypotheses. As a whole, the program should teach optimal utilization of population-level data resources. For instance, researchers should be aware that with large data, many associations could become statistically significant, but they may not be clinically relevant. Further, though also present in smaller data resources, bias becomes an increasingly important issue when using population-scale data. This training program not only helps trainees identify these areas of concern, but also gives them the tools to address them.

In the case of ADRD, trainees with expertise in neuroimaging will need to understand population sciences to identify the population at risk of ADRD; use data science to quantify the impact of the multi-faceted aspects of aging phenotype on neuroimaging outcomes, and acquire content expertise in gerontology. Following this general model, the T32 training in PNA provides neuroimaging researchers with training in three main areas. First, trainees learn population science methods to design studies to identify at risk individuals; this includes learning to plan and evaluate population sampling, interpret biases, and interpret change in measures. Second, training in data sciences provides the knowledge necessary to integrate large amounts of data, test for moderation (interaction), confounding and mediation effects among others. Lastly, training in gerontology provides content area expertise, and specifically in age-related basic biological and physiological processes underlying ADRD; this foundational knowledge helps explain how alterations in physiology with aging cause dysregulated systems and diseases which in turn can drive ADRD risk. Of course, trainees also need to develop a toolkit of professional skills common to most investigators (see section on competencies below).

## Define competencies

An effective training program must have clear objectives and measurable competencies that all trainees must achieve in order to become successful investigators in the topic area. Some objectives and competencies will be specific to the training program while others are pertinent to all investigators. The PNA program as 4 objectives: (1) Recruit and train highly qualified scholars from diverse backgrounds into the field of PNA; (2) Provide foundational knowledge in PNA; (3) Train in conducting rigorous research in PNA; (4) Foster professional development to lead multi-disciplinary research teams in PNA.

Table 2 offers a summery of competencies required for PNA trainees upon completion of the training program. Trainees should be able to integrate key concepts and techniques from epidemiological, neuroimaging, and data science methods in cross-discipline collaborations. Their goal is to design, implement, and interpret research in neuroimaging in ADRD to answer questions pertaining etiology and pathophysiology. The successful investigator will be able to integrate these concepts and approaches into novel research questions and rigorous research projects. Overall, population neuroimaging researchers will be able to conduct earlier and more precise characterizations of the neurocognitive phenotypes of ADRD. This in turn can improve causal inference in observational epidemiological studies of dementia, enable development of biomarkers for early case detection, and permit accurate monitoring of response to therapy. Examples of novel research projects undertaken by the PNA trainees to date include:

- Applying aspects of data science (e.g., bioinformatics, and biostatistics) to neuroimaging research in AD;
- Developing neuroimaging research on health disparities in AD/ADRD;
- Cross-national dementia research using harmonized data on neuroimaging and cognitive function;
- Analyses of multi-level data (e.g., biological, behavioral, social, environment) from publicly available and/or deidentified sources.

**Table 2:** Competencies of population neuroimaging researchers expected upon completion of the training program in Population Neuroscience of Alzheimer's Disease (PNA).

**Scientific competencies**	**Professional Competencies**
• Interpret/critique results of neuroimaging studies of AD conducted in epidemiological cohorts. • Apply modern epidemiologic concepts and techniques to successfully identify at risk populations, minimize bias, and account for multifactorial effects in their own study. • Understand how demographic and health characteristics of older populations depend on the study setting (e.g., community, research clinic), and modify study designs accordingly. • Acquire in-depth knowledge of the complex inter-relationships between demographic, multi-morbidities and genetic/epigenetic factors contributing to AD, • Apply data driven approaches to understand the role of the exposome in AD.	• Work with a multidisciplinary team. • Effectively communicate their results *via* public speaking, writing manuscripts/grants; • Deal with the media to disseminate research results. • Navigate the professional environment. • Plan, submit and manage research grants

Once the key competencies have been established, the next two steps are: building collaborations with other departments and schools and creating a program-specific curriculum with clear selection criteria and milestones.

## Build collaborations with departments from relevant disciplines

Based on the set of competencies, program leaders at a given institution should identify individual investigators in participating disciplines/departments and relevant formal coursework at that organization.

The epidemiology training faculty contribute expertise in advanced epidemiological study design and methods. This group of faculty appreciates that epidemiological approaches to understanding brain diseases have to be integrated with advanced methodologies to quantify the CNS. The faculty with expertise in Data Science teach trainees how to integrate quantitative approaches with an understanding of the underlying conditions and with rigorous study design. Their work pertains to machine learning, neural network modeling, and non-linear dynamics approaches to understanding brain and behavior. The neuroscience training faculty apply a variety of measures in their research, including neuroanatomical and neuroimaging methods to assess neural structure and connection pathways, opto-genetic methods to link molecular, neural and behavioral levels of analysis, neurophysiological methods to assess neural function in awake, behaving animals, and neuropharmacological methods to measure and manipulate neurotransmitter systems. The training faculty members with focus on gerontology offer expertise on the spectrum of “normal” and accelerated age-related physiologic change, the biological processes underlying aging, and new potential targets for prevention of age-related chronic diseases and functional impairment. Collectively, the PNA faculty have programs of research involving human and non-human normative and disordered behaviors of aging, links to corresponding neural structure, function and behavior, analytical strategies to integrate these data, and implications for optimal study design.

## Develop curriculum and experiential learning to achieve competencies

**Figure 2:**
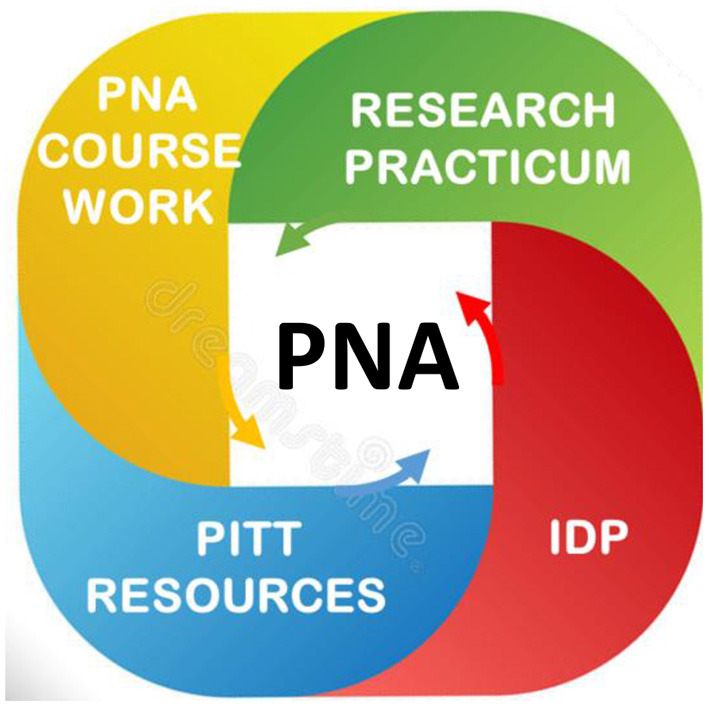
The curriculum of the training program in population neuroscience of Alzheimer's Disease (PNA). The PNA curriculum is articulated in 4 main components. Individual Development Plan (IDP). The mentoring team sets up milestones and objectives, with the goal to identify the curriculum tailored to each trainee's needs, closely mentored, and frequently evaluated. It identifies professional development activities targeted to the trainee's goal, as well clinical rotations in neurocognitive geriatric assessments and dementia adjudication at the ADRC. PNA coursework: provides foundational knowledge in population science, neuroscience, geroscience and data science. It teaches practical skills (review of peers' work; write and present a novel independent project). PNA Research Practicum: provides skills in scientific reasoning; research design; quantitative approaches, data analysis/interpretation, write and submit manuscripts, submit grant (required for postdocs). Pitt Resources: PhD programs in Epidemiology, Neuroscience, Psychology, Biostatistics, Biomedical Informatics offer relevant courses. Many centers and Institutes offer space for research, pilot funds, and professional development activities: ADRC; Center for Neural basis of Cognition; Aging Institute; CTSI; Brain Institute, Office Academic Career Development; Teaching Institute.

Based on the set of required competencies, program leaders should search for relevant existing formal coursework at their institution, and then plan a new curriculum to fill the gaps. To the extent needed, arrangements should be made early to create access to the coursework and to work out any other interdepartmental/ interschool agreements. The PNA curricular strategy consists of *4 components*, illustrated in Figure 2.

Individual development plan (IDP) and multidisciplinary mentoring teams. Each trainee is assigned a primary mentor and a mentoring team based on the specific disciplinary expertise that is likely to be relevant. Post-doctoral trainees enter the program with their primary mentor already identified. These mentoring teams are designed to assure that the trainee gains a strong multidisciplinary perspective on important research gaps and on the advantages and disadvantages of various design, implementation and interpretation challenges. With team input, each trainee selects one or more research questions where they will take a lead in some aspects of implementation as well as analyses and manuscript preparation. All postdoctoral and some pre-doctoral trainees are required to develop and submit research proposals for independent funding. The IDP is crafted by the trainee and the mentoring team to custom-tailor each trainee's project, ensure synergy among coursework, practicum, and other professional development activities, set milestones, and monitor progress, meeting at least twice a year with the mentoring team. Like all graduate and post-graduate research training, much of the most effective learning comes from experiences outside the classroom. The IDP includes professional development activities; trainees are required to attend, lead, and propose multi-disciplinary activities ranging from journal clubs, seminars, workshops, Science blitz, Book clubs and more. Some are specific to the PNA, and many are hosted in other departments, thus these activities continually infuse an integrative research perspective into PNA trainee experiences, facilitate inter-disciplinary networking and provide first-hand experience to bridge between separate disciplines and department-centric cultures. A key part of the process is to encourage all trainees to provide constructive feedback to one another. To increase exposure to the PNA disciplines, trainees are required to attend at least 1 seminar/ month outside their home department. Training in research conduct and reporting ensures that trainees are ethically and morally responsible scientists who conduct and report rigorous, transparent, and reliable research.

The PNA coursework provides foundational knowledge in PNA and opportunities to learn concrete skills (data collection, data harmonization, data management, statistics, research design, grant review), methods to enhance reproducibility, scientific writing, and multimodal methodologies to measure age-related brain changes (neuroimaging, cognitive, and post-mortem assessments). In addition, existing graduate programs offer relevant courses in complementary disciplines. The course in “Population Neuroscience” familiarizes the students with existing neuro-epidemiological studies and publicly available datasets. Through the “Introduction to Multimodal Neuroimaging” course trainees learn the principles of neuroimaging techniques, including data modeling and visualization, to identify the main principles of paradigm design, collection and processing of neuroimaging data, as well as to critically evaluate strengths and limitations of each modality. The content of this course is used to complete the “Writing in Population Neuroscience” course; the purpose of this course is to write a manuscript or grant proposal on a theme related to population neuroscience. Existing coursework in gero-science, epidemiology and data science address perspectives on study design, multiple comorbidities of aging affecting ADRD, and advanced analytical methods.

The research practicum complements the PNA courses, it extends the trainees' projects *via* mentored rotations in study design, scientific reasoning, data collection, analysis and interpretation and reporting of their results. In the research practicum, trainees apply concepts taught in class. All trainees rotate through several participating laboratories and research programs where they are exposed to a range of ongoing processes and approaches, they can use their own newly collected data, as well as existing neuroepidemiological datasets from multi-center national and international studies. Trainees use PNA courses and research practicum to publish 1^st^-authored manuscripts; postdocs submit grant proposals.

Extensive resources at the University of Pittsburgh: The PNA program is supported by three Schools and eight departments: Public Health (Departments of Epidemiology, and Biostatistics), Medicine (Departments of Psychiatry, Neurology, Geriatric Medicine, Biomedical Informatics), Arts and Science (Departments of Neuroscience, Psychology). The PNA mentors are faculty with primary appointments in one of these departments, secondary appointments in other departments and affiliations with at least one of these graduate programs. Collectively, PNA mentors provide PNA trainees with 40+ neuroepidemiological studies (ongoing and existing studies) both to carry out secondary data analyses and to design novel original ancillary pilot studies. The PNA program leverages numerous transdisciplinary Centers of Excellence and Institutes: the Alzheimer's Disease Research Center, the Center for the Neural Basis of Cognition, the Brain Institute, the Center for Aging, Population and Health, and the Aging Institute. These Centers of Excellence and Institutes provide space for clinic exams, brain scanners, biomarkers labs, funds for pilot data collection and analyses, and additional educational opportunities (seminars, journal clubs, workshops). The Office for Academic Career Development offers seminars on grant writing / professional development. The Provost office supports recruitment of trainees from diverse backgrounds.

## Identify selection criteria

Primary selection criteria to enter the PNA program are: (a) prior research experience (>2 for pre-doctoral students, > 4 for post-doctoral scholars), (b) a research plan that is aligned with the PNA thematic area, and includes at least one PNA mentor; (c) a clear commitment to expand one's foundational knowledge basis; and (d) >2 first authored publication for post-docs. Additional criteria are: strong letters of recommendation, and a plan to submit an independent grant proposal. Although a GPA> 3.6 was initially one of the selection criteria, it was later removed, because of evidence it may affect equitable appointments (Weiner, 2014; Hall et al., 2017). Nobody is barred from applying on the basis of their home training program or affiliations of their primary faculty advisor. However, as part of the application, trainees must indicate a commitment to the goals and requirements of the PNA program, provide evidence that they have approval from their home training program, and demonstrate the support of their primary faculty advisor. The same level of commitment is expected for post-doc applicants. Finally, additional factors are considered, such as the representation of the participating departments, representation of underrepresented minorities and trainees with disabilities, and the balance across our thematic research areas. As result of the PNA recruitment efforts and selection criteria, in its first few years the PNA program recruited a diverse cohort in terms of scientific background and identity/demographics, with 50% URG (higher than the proportion of 13% at Pitt), all with more than 3 years of research experience, many with publications and experience publishing grants.

## Establish milestones

Because of its multi-disciplinary nature, PNA training must be carefully coordinated among mentors from each discipline. After an initial orientation period, each trainee prepares an Individual Development Plan (IDP) based on the framework suggested by the NIH-funded Institute for Clinical Research and Education. This plan identifies short- and long-term career goals and reviews the program competencies to determine which the trainee has already accomplished and which are to be developed during training. The trainee also lays out timelines for formal coursework and experiential research activities, including manuscripts and grant proposals. Biannual review of the IDP with the mentors and the PIs of the T32 serves to review timelines, and to assess whether the trainee is on track to meet their goals. IDPs are designed to be flexible and adapt to changing needs and opportunities. Trainees are required to write at least one first-authored manuscript that clearly demonstrates that they have achieved PNA competencies. They are trained in grant writing and are encouraged to write and submit one grant proposal as principal investigator.

## Lessons learned

Much of what was initially envisioned has worked well. In its first 4 years, the PNA enrolled 7 predoctoral trainees and 3 postdoctoral trainees from diverse disciplinary backgrounds including psychology, neuroscience, and epidemiology. Among the six trainees who have been in the program for more than 1 years, all completed core and interdisciplinary coursework, and seminars, and produced novel research findings and generate novel ideas based on a multidisciplinary perspective of ADRD, as demonstrated by their scientific productivity as PNA trainees (Alber et al., 2019; Shaaban et al., 2019a,b,c, 2021, 2022; Cui et al., 2020; Johnson et al., 2020; Lin et al., 2020; Ly et al., 2020a,b, 2021; Shaaban and Molad, 2020; Tian et al., 2020, 2022; Aghjayan et al., 2021; Chahine et al., 2021; Ehrenkranz et al., 2021; Freed et al., 2021; Sprague, 2021; Sprague et al., 2021a,b; Yu et al., 2021; Donofry et al., 2022; Mielke et al., 2022; Silva et al., 2022). Four of 6 trainees submitted an NIH grant while in training, showing that using PNA resources enables them to conduct independent and rigorous research PNA trainees are committed to following a career in academic research.

There are signs that these efforts have not only affected trainee's careers, but are also stimulating cultural and scientific change within the partnering departments. In several instances, the trainees themselves have initiated new inter-departmental and inter-school collaborations. For example, faculty from experimental behavioral research and clinical neuroscience programs now collaborate with faculty in population science. Moreover, in the planning of the PNA curriculum, new collaborative efforts have formed between Epidemiology, Biostatistics, the School of Information Science, and Biomedical Informatics. These collaborations have translated into new didactic modules for PNA trainees, including a PNA “traveling seminar,” consisting of guest lectures in existing neuroscience courses and seminars series, and in data science webinars co-sponsored by the PNA and established institutes.

Positioning the PNA training program in a milieu as rich as the one at the University of Pittsburgh, also has challenges. Our faculty face difficulties finding time to engage in PNA activities across departments and schools. The traveling seminars will make it easier for faculty to participate.

Feedback from trainees expressed strong support for the PNA-specific Training components, including the course requirements, weekly works in progress, the mentoring teams, and the experiential rotations. They appreciate opportunities to interact and work with researchers across many disciplines. As with any new program, there are many lessons learned. For example, some trainees must take quite a lot of formal coursework beyond that of their home department degree (for example, doctoral students in Epidemiology take classes in neuroscience); that can eat into time to carry out research projects in a timely fashion. To address this concern, trainees have the option to extend training to 3 years, based on the recommendation of the primary mentor.

Finally, recruitment efforts must adapt to this generation's preferred communication media. In addition to traditional recruitment routes through national outreach to Schools, Conferences and National Associations specialized in disciplines related to our program, the PNA program has a social media presence on Twitter and Facebook. As a result of all these new social media strategies, a new challenge is arising; where to find the time, effort, and communication skills to sustain social media platforms.

## Conclusion

Population neuroimaging is a vigorous and exciting field with exceptional opportunities for groundbreaking discoveries in the future. The type of multidisciplinary training described here can be applied to many other programs related to population neuroimaging beyond ADRD. Population neuroimagers have an extraordinarily timely opportunity to invest in innovative training models to foster the development of a new group of multidisciplinary translational researchers. Multidisciplinary training programs are aligned with both national and institutional mandates for translational and multidisciplinary research. Such programs have potential to strengthen and foster a culture dedicated to comprehensive multidisciplinary training in population neuroimaging.

## Author contributions

The author confirms being the sole contributor of this work and has approved it for publication.

## Funding

The work reported here was supported in part by grant # 1 T32 AG055381, from the National Institute of Aging, National Institutes of Health, United States Department of Health and Human Services.

## Conflict of interest

The author declares that the research was conducted in the absence of any commercial or financial relationships that could be construed as a potential conflict of interest.

## Publisher's note

All claims expressed in this article are solely those of the authors and do not necessarily represent those of their affiliated organizations, or those of the publisher, the editors and the reviewers. Any product that may be evaluated in this article, or claim that may be made by its manufacturer, is not guaranteed or endorsed by the publisher.
